# Triterpenoid saponins and ferroptosis: A membrane centered perspective

**DOI:** 10.17179/excli2026-9566

**Published:** 2026-06-12

**Authors:** Tae Kyung Hyun

**Affiliations:** 1 Department of Industrial Plant Science and Technology, College of Agriculture, Life and Environment Sciences, Chungbuk National University, Cheongju 28644, Republic of Korea

## ⁯⁯

Ferroptosis is an iron-dependent form of regulated cell death driven by phospholipid peroxidation and membrane damage (Yang and Gao, 2026[9]). Unlike apoptosis and necrosis, ferroptosis is triggered by impaired detoxification of lipid hydroperoxides following disruption of glutathione peroxidase 4 (GPX4), resulting in uncontrolled membrane lipid oxidation (Li et al., 2020[4]). Recent studies indicate that ferroptotic susceptibility is strongly influenced by phospholipid composition, iron dependent redox activity, and membrane vulnerability to lipid peroxidation (Cui and Ye, 2026[1]; Yang and Gao, 2026[9]). Importantly, many ferroptosis associated pathways converge on membrane lipid peroxidation, suggesting that membrane organization may regulate ferroptotic sensitivity. In this context, membrane active natural compounds may represent previously underrecognized modulators of ferroptotic vulnerability.

Triterpenoid saponins are a structurally diverse class of amphiphilic plant metabolites composed of hydrophobic triterpene backbones linked to hydrophilic sugar moieties. Owing to their intrinsic affinity for biological membranes, these compounds can alter membrane organization, lipid dynamics, and membrane-associated redox processes (Zhong et al., 2025[10]). Accordingly, triterpenoid saponins have attracted attention for their broad pharmacological activities, including anticancer, immunomodulatory, and anti-inflammatory effects (Ouyang et al., 2025[6]). However, the mechanisms underlying their effects on regulated cell death pathways remain incompletely understood.

A major unresolved issue is that many cellular responses associated with triterpenoid saponin-induced cytotoxicity overlap with generalized oxidative injury, making it difficult to distinguish ferroptotic signaling from secondary oxidative damage (Podolak et al., 2023[7]). Nevertheless, several triterpenoid saponins have been reported to modulate key ferroptosis-related regulators, including the cystine/glutamate antiporter-glutathione-GPX4 axis, Nrf2 signaling, and p53-dependent pathways (Supplementary Table 1). For example, ginsenoside Rg3 has been shown to induce ferroptosis in hepatic stellate cells through regulation of the miR-6945-3p/DNMT3B/ACSL4 axis (Hu et al., 2024[2]), thereby alleviating liver fibrosis, and to inhibit melanoma progression by inducing ferroptosis through the p53/SLC7A11/GPX4 axis (Ma et al., 2025[5]). Similarly, saikosaponin D promotes ferroptosis in bladder cancer cells (Huang et al., 2026[3]), but alleviates ferroptosis-associated tissue injury in sepsis-induced acute lung injury through activation of Nrf2 signaling (Song et al., 2025[8]). These observations suggest that triterpenoid saponins function as context-dependent modulators rather than simple ferroptosis inducers or inhibitors. Given that ferroptosis is fundamentally linked to membrane lipid peroxidation, the membrane-active properties of saponins may explain their context-dependent effects on ferroptotic susceptibility. This observation suggests that membrane organization and the lipid microenvironment may serve as important determinants of ferroptotic sensitivity.

## Declaration

### Conflict of interest

The author declares no conflict of interest.

### Artificial Intelligence (AI) - assisted technology

During the preparation of this manuscript, the author utilized ChatGPT (version GPT-5) for language refinement.

## Supplementary Material

Supplementary information
