# Supplementary material for: Triterpenoid saponins and ferroptosis: A membrane centered perspective
Source: EXCLI J. 2026 Jun 12;25:822–3. doi: 10.17179/excli2026-9566 (PMC13312795; doi:10.17179/excli2026-9566)
Supplement: Supplementary information [file EXCLI-25-822-s-001.pdf]

*EXCLI Journal 2026;25:822-823 – ISSN 1611-2156*
*Received: May 19, 2026, accepted: May 22, 2026, published: June 12, 2026*

## Supplementary information to:

### Letter to the editor:

# TRITERPENOID SAPONINS AND FERROPTOSIS: A MEMBRANE CENTERED PERSPECTIVE

Tae Kyung Hyun*

Department of Industrial Plant Science and Technology, College of Agriculture, Life and Environment Sciences, Chungbuk National University, Cheongju 28644, Republic of Korea

* **Corresponding author:** Tae Kyung Hyun, Department of Industrial Plant Science and Technology, College of Agriculture, Life and Environment Sciences, Chungbuk National University, Cheongju 28644, Republic of Korea; E-mail: taekyung7708@chungbuk.ac.kr; Phone: +82-43-261-2520; Fax: +82-43-271-0413

https://dx.doi.org/10.17179/excli2026-9566

This is an Open Access article distributed under the terms of the Creative Commons Attribution License (https://creativecommons.org/licenses/by/4.0/).

**Supplementary Table 1:** Context-dependent modulation of ferroptosis by triterpenoid saponins

| Function | Compounds | Source | Key finding | Ref. |
|---|---|---|---|---|
| Inhibitor | Platycodin D | *Platycodon grandiflorus* | Platycodin D attenuated ferroptosis in granulosa cells by targeting the CD44/SLC7A11 axis to activate system $Xc^{-}$, thereby alleviating pathological damage associated with polycystic ovary syndrome. | Ji et al., 2024 |
| | Ginsenoside Rg1 | Ginseng | In rats with sepsis induced myocardial injury, ginsenoside Rg1 alleviated cardiac damage by regulating the FAK/AKT-FOXO3A signaling pathway, thereby suppressing ferroptosis associated oxidative stress and restoring antioxidant defenses. | Lin et al., 2024b |
| | | | Ginsenoside Rg1 alleviated ferroptosis in cigarette smoke induced chronic obstructive pulmonary disease by suppressing endoplasmic reticulum stress through regulation of the PERK/ATF4 axis. | Tan et al., 2024 |
| | | | Ginsenoside Rg1 alleviated sepsis induced acute kidney injury by suppressing ferroptosis in renal tubular epithelial cells through the ferroptosis suppressor protein 1–CoQ10–NAD(P)H pathway. | Guo et al., 2024 |
| | | | Ginsenoside Rg1 counteracted diabetic osteoporosis by suppressing ferroptosis through modulation of mitochondrial membrane potential and upregulation of GPX4 expression. | Chen et al., 2026 |
| | Ginsenoside Rh2 | Ginseng | Ginsenoside Rh2 suppressed ferroptosis in ulcerative colitis by upregulating microRNA-125a-5p, thereby targeting specificity protein 1. | Zhao et al., 2024b |
| | | | Ginsenoside Rh2 exerted neuroprotective effects in Alzheimer's disease models by modulating the Nrf2/GPX4 axis, thereby suppressing ferroptosis, oxidative stress, and neuroinflammation while improving cognitive function. | Meng et al., 2026 |
| | Ginsenoside Re | Ginseng | Ginsenoside Re alleviated oxidative stress and ferroptosis in pulmonary fibrosis mice through regulation of the Nrf2/Keap1/GPX4 axis. | Lin et al., 2024a |
| | | | Ginsenoside Re increased GPX4 expression in EA.hy926 cells and alleviated homocysteine induced endothelial ferroptosis. | Li et al., 2025 |

EXCLI Journal 2026;25:822-823 – ISSN 1611-2156
Received: May 19, 2026, accepted: May 22, 2026, published: June 12, 2026

| Function | Compounds | Source | Key finding | Ref. |
| --- | --- | --- | --- | --- |
| Inhibitor | Ginsenoside Rg3 | Ginseng | Ginsenoside Rg3 inhibited melanoma progression by inducing ferroptosis through the p53/SLC7A11/GPX4 signaling pathway. | Ma et al., 2025 |
| | | | Ginsenoside Rg3 inhibited ferroptosis in nucleus pulposus cells through regulation of PRKAA2 expression. | Xu et al., 2025 |
| | | | Ginsenoside Rg3 attenuated myocardial ischemia/reperfusion induced ferroptosis through the Keap1/Nrf2/GPX4 signaling pathway. | Zhong et al., 2024 |
| | | | Ginsenoside Rg3 ameliorated di-(2-ethylhexyl) phthalate induced spermatogenic impairment by attenuating ferroptosis and restoring Sertoli cell secretory function. | Gao et al., 2026 |
| | Saikosaponin A | *Bupleurum falcatum* | Saikosaponin A alleviated seizures and improved cognitive function in temporal lobe epilepsy by suppressing IL-17/Akt/ERK mediated ferroptosis in hippocampal neurons. | Li et al., 2026 |
| | Saikosaponin D | *Bupleurum falcatum* | Saikosaponin D ameliorated sepsis induced acute lung injury by preserving alveolar epithelial barrier integrity and suppressing ferroptosis through activation of Nrf2 signaling. | Song et al., 2025 |
| | | | Saikosaponin D alleviated liver ischemia reperfusion injury by suppressing mitophagy associated ferroptosis through the STAT3/PINK1 pathway. | Liu et al., 2026 |
| Inducer | Platycodin D | *Platycodon grandiflorus* | Platycodin D promoted ferroptosis in prostate cancer cells via the METTL16/m6A/NUPR1 axis, thereby enhancing the therapeutic efficacy of docetaxel. | Sun et al., 2025 |
| | | | Platycodin D inhibited non-small cell lung cancer bone metastasis by inducing ferroptosis through the miR-877-3P/PNMA5 regulatory axis, a key regulator of osteoclast differentiation and osteolytic lesion formation within the metastatic microenvironment. | Xie et al., 2025 |
| | Ginsenoside Rh2 | Ginseng | Ginsenoside Rh2 upregulated IRF1 expression, leading to suppression of SLC7A11, thereby promoting ferroptosis and inactivation of hepatic stellate cells. | Lang et al., 2023 |
| | | | Ginsenoside Rh2 targeted prostate cancer by inducing mitochondrial damage, thereby activating PINK1/Parkin mediated mitophagy and ferroptosis. | He et al., 2025 |
| | Ginsenoside Rh3 | Ginseng | Ginsenoside Rh3 induced pyroptosis and ferroptosis in colorectal cancer cells through the STAT3/p53/NRF2 signaling axis. | Wu et al., 2023 |

| Function | Compounds | Source | Key finding | Ref. |
|---|---|---|---|---|
| Inducer | Ginsenoside Rh4 | Ginseng | Ginsenoside Rh4 induced ferroptosis in lung cancer by inhibiting the KEAP1/NRF2/HO-1 pathway and remodeling the gut microbiota to increase butyrate production, thereby enhancing ferroptotic sensitivity through ATF3 activation and GPX4 suppression. | Zhu et al., 2026 |
| | | | Ginsenoside Rh4 enhanced the ferroptotic sensitivity of Renal cell carcinoma cells by inhibiting the NRF2 pathway and suppressing antioxidant defense systems. | Zhao et al., 2024a |
| | Ginsenoside Rg3 | Ginseng | Ginsenoside Rg3 promoted ferroptosis in hepatic stellate cells and alleviated liver fibrosis through miR-6945-3p mediated inhibition of DNMT3B and subsequent suppression of ACSL4 methylation. | Hu et al., 2024 |
| | | | Ginsenoside Rg3 inactivated the circFOXP1/miR-4477a/PD-L1 signaling axis to enhance CD8+ T cell immune function, thereby inducing ferroptosis and apoptosis in gallbladder cancer cells. | Ye et al., 2024 |
| | Saikosaponin A | *Bupleurum falcatum* | Saikosaponin A attenuated osteoclastogenesis and bone loss by inducing osteoclast ferroptosis through inhibition of the Nrf2/SLC7A11/GPX4 signaling pathway. | Li et al., 2024 |
| | | | Saikosaponin A induced ferroptosis in hepatocellular carcinoma cells through endoplasmic reticulum stress mediated ATF3 activation. | Lan et al., 2023 |
| | Saikosaponin D | *Bupleurum falcatum* | Saikosaponin D inhibited the malignant phenotype of bladder cancer by suppressing the PI3K/AKT signaling pathway and promoting ferroptosis. | Huang et al., 2026 |

### *Conflict of interest*

The author declares no conflict of interest.

### *Artificial Intelligence (AI) – assisted technology*

During the preparation of this manuscript, the author utilized ChatGPT (version GPT-5) for language refinement.

## REFERENCES

Chen M, Zheng H, Bai R, Huang Y, Pang G, Zhu H, et al. Ginsenoside Rg1 antagonizes diabetic osteoporosis by regulating ferroptosis via mitochondrial membrane potential in H-type vascular endothelial cells. Front Aging. 2026;7:1736263.

Gao XF, Ma N, Zhang YZ, Zhang J, Liu Z, Zhu HY, et al. Ginsenoside Rg3 ameliorates di-(2-ethylhexyl) phthalate-induced spermatogenic impairment via attenuating ferroptosis and restoring sertoli cell secretory dysfunction. J Environ Sci. 2026;165:877–890.

Guo J, Chen L, Ma M. Ginsenoside Rg1 Suppresses Ferroptosis of Renal Tubular Epithelial Cells in Sepsis-induced Acute Kidney Injury via the FSP1-CoQ10-NAD(P)H Pathway. Curr Med Chem. 2024;31:2119–2132.

He Z, Shi J, Zhu B, Tian Z, Zhang Z, Zhang C. Ginsenoside Rh2 repressed the progression of prostate cancer through the mitochondrial damage induced by mitophagy and ferroptosis. Front Oncol. 2025;15: 1633891.

EXCLI Journal 2026;25:822-823 – ISSN 1611-2156
Received: May 19, 2026, accepted: May 22, 2026, published: June 12, 2026

Hu Y, Lang Z, Li X, Lin L, Li Y, Zhang R, et al. Ginsenoside Rg3 promotes hepatic stellate cell ferroptosis by epigenetically regulating ACSL4 to suppress liver fibrosis progression. Phytomedicine. 2024;124: 155289.

Huang H, Guo L, Sun H, Liu Y, Zhang J, Tang W, et al. Saikosaponin D inhibits bladder cancer growth and enhances the synergistic antitumor effect of gemcitabine by targeting PI3K/AKT-mediated ferroptosis. Biochem Biophys Res Commun. 2026;816:153709.

Ji R, Wang S, Chen X, Yang Z, Zhang Z, Bao S, et al. Platycodin D ameliorates polycystic ovary syndrome-induced ovarian damage by upregulating CD44 to attenuate ferroptosis. Free Radic Biol Med. 2024;224: 707–722.

Lan T, Wang W, Zeng XX, Tong YH, Mao ZJ, Wang SW. Saikosaponin A triggers cell ferroptosis in hepatocellular carcinoma by inducing endoplasmic reticulum stress-stimulated ATF3 expression. Biochem Biophys Res Commun. 2023;674:10–18.

Lang Z, Yu S, Hu Y, Tao Q, Zhang J, Wang H, et al. Ginsenoside Rh2 promotes hepatic stellate cell ferroptosis and inactivation via regulation of IRF1-inhibited SLC7A11. Phytomedicine. 2023;118:154950.

Li PP, Wang WW, Wang JT, Xu ZA, Ji XF, Guan L, et al. Anti-epileptic effect of saikosaponin a by inhibiting ferroptosis via the IL-17/Akt/ERK signaling pathway in the temporal lobe epilepsy model. Naunyn Schmiedebergs Arch Pharmacol. 2026;399:2565–2575.

Li S, Zhao C, Yu S, Yang K, Zhang S, Liu S. Ginsenoside Re attenuates homocysteine-induced endothelial cell ferroptosis through upregulation of GPX4/xCT signaling. Exp Ther Med. 2025;31:27.

Li TQ, Liu Y, Feng C, Bai J, Wang ZR, Zhang XY, et al. Saikosaponin A attenuates osteoclastogenesis and bone loss by inducing ferroptosis. Front Mol Biosci. 2024;11:1390257.

Lin H, Wen Z, Feng L, Chen X, Song Y, Deng J. Ginsenoside Re Alleviates Oxidative Stress Damage and Ferroptosis in Pulmonary Fibrosis Mice by Regulating the Nrf2/Keap1/GPX4 axis. Int J Drug Discov Pharmacol. 2024a;3:100025.

Lin LQ, Mao FK, Lin J, Guo L, Yuan WR, Wang BY. Ginsenoside Rg1 induces ferroptosis by regulating the focal adhesion kinase/protein kinase B-forkhead box O3A signaling pathway and alleviates sepsis-induced myocardial damage. J Physiol Pharmacol. 2024b;75: 389–401.

Liu X, Xu Y, Yu X, Feng X, Han Y, Cai H, et al. Saikosaponin D Alleviates Liver Ischemia–Reperfusion Injury by Inhibiting Mitophagy-Associated Ferroptosis via the STAT3/PINK1 Pathway. Phytother Res. 2026. Available from: https://onlinelibrary.wiley.com/doi/10.1002/ptr.70333

Ma A, Zhu S, Yao X, Chen Y, Yao J, Shen M, et al. Ginsenoside Rg3 inhibits melanoma progression by inducing ferroptosis via the p53/SLC7A11/GPX4 pathway. J Adv Res. 2025:S2090-1232(25)00864-1.

Meng Q, Li J, Xu G, Zhang W, Cao R, Cai K. Ginsenoside Rh2 Alleviates Alzheimer Disease Models via Effects on Ferroptosis-Related Neuroinflammation. J Biochem Mol Toxicol. 2026;40:e70860.

Song L, Tao Y, Lu G, Wu C. Saikosaponin D ameliorates sepsis-induced acute lung injury by maintaining alveolar epithelial barrier integrity and inhibiting ferroptosis via Nrf2/HO-1 pathway. Inhal Toxicol. 2025; 37:195–207.

Sun C, Sun X, Chen Y, Wu Y, Xiang C, Wu S. Platycodin D-mediated METTL16 downregulation promotes docetaxel treatment of prostate cancer by regulating ferroptosis. BMC Cancer. 2025;25:1042.

Tan W, Liang Z, Tan X, Tan G. Ginsenoside Rg1 improves cigarette smoke-induced ferroptosis in COPD by regulating PERK/ATF4 axis to inhibit endoplasmic reticulum stress. Biochem Biophys Res Commun. 2024;739:150946.

Wu Y, Pi D, Zhou S, Yi Z, Dong Y, Wang W, et al. Ginsenoside Rh3 induces pyroptosis and ferroptosis through the Stat3/p53/NRF2 axis in colorectal cancer cells. Acta Biochim Biophys Sin. 2023;55:587–600.

Xie Z, Zhou Z, Wang S, Wang Y, Cheng K, Lin J, et al. Platycodin D inhibits non-small cell lung cancer bone metastasis by inducing ferroptosis through miR-877-3P/PNMA5 regulatory axis. Phytomedicine. 2025; 149:157534.

Xu W, Geng F, Zhang K, Wang Y. Targeting Ferroptosis With Ginsenoside Rg3 Alleviates Intervertebral Disc Degeneration. Orthop Surg. 2025;17:2960–2972.

Ye Z, Ding J, Huang J, Hu Z, Jin F, Wu K. Ginsenoside Rg3 activates the immune function of CD8+ T cells via circFOXP1-miR-4477a-PD-L1 axis to induce ferroptosis in gallbladder cancer. Arch Pharm Res. 2024;47: 793–811.

Zhao H, Ding R, Han J. Ginsenoside Rh4 Facilitates the Sensitivity of Renal Cell Carcinoma to Ferroptosis via the NRF2 Pathway. Arch Esp Urol. 2024a;77:119–128.

*EXCLI Journal 2026;25:822-823 – ISSN 1611-2156*
*Received: May 19, 2026, accepted: May 22, 2026, published: June 12, 2026*

Zhao X, Yuan W, Yang L, Yan F, Cui D. Ginsenoside Rh2 suppresses ferroptosis in ulcerative colitis by targeting specific protein 1 by upregulating microRNA-125a-5p. Eur J Med Res. 2024b;29:450.

Zhong G, Chen J, Li Y, Han Y, Wang M, Nie Q, et al. Ginsenoside Rg3 attenuates myocardial ischemia/reperfusion-induced ferroptosis via the keap1/Nrf2/GPX4 signaling pathway. BMC Complement Med Ther. 2024;24:247.

Zhu Q, Xu W, Yang G, Gao Y, Zhao Y, Zhao Z, et al. Ginsenoside Rh4 Triggers Ferroptosis in Lung Cancer: Targeting KEAP1/NRF2/HO-1 and Remodeling Gut Microbiota for Butyrate-Mediated ATF3 Activation. Int J Mol Sci. 2026;27:2703.
